# Emerging Therapeutic Strategies for Child and Adolescent Obesity: A Narrative Review

**DOI:** 10.3390/nu18203449

**Published:** 2026-10-21

**Authors:** Tomaso Oliaro, Federico Amianto

**Affiliations:** 1 Department of Public Health and Pediatric Sciences, University of Turin, 10126 Turin, Italy; 2 Neurosciences Department, University of Turin, 10126 Turin, Italy

**Keywords:** child obesity, multidisciplinary approach, family treatment, behavioral treatment, drug treatment, long-term effects

## Abstract

Background/Objectives: Childhood obesity is a chronic, multifactorial disease requiring attention to biological, behavioral, psychological, family, and socioeconomic factors. This narrative review examined the integration of lifestyle, family-based, psychological, and pharmacological approaches, focusing on treatment engagement, eating disorders, attention-deficit/hyperactivity disorder (ADHD), and treatment durability. Methods: A single search interface covering PubMed/MEDLINE, Scopus, and Web of Science (the University of Turin library discovery platform) was used to identify studies published primarily from 2010 to August 2026. Pediatric trials, observational studies, systematic reviews, meta-analyses, guidelines, and public-health reports were prioritized. Results: Intensive family-based, multicomponent behavioral treatment remains the best-supported foundation of care, although psychological outcomes are infrequently assessed. Psychiatric comorbidity is heterogeneous; loss-of-control eating, emotional symptoms, ADHD, and executive-function difficulties may impair engagement in selected patients, and untreated ADHD has been associated with a slower BMI response to weight management. Professionally supervised obesity treatment containing a dietary component did not, on average, worsen eating psychopathology and reduced several related symptoms. Family participation improved outcomes, whereas logistical, socioeconomic, and psychological barriers reduced attendance. Across randomized trials, adding anti-obesity medication to behavioral treatment produced an additional, although on average modest, reduction in BMI. Semaglutide, liraglutide, and phentermine/topiramate produced larger numerical reductions than older agents in separate trials, but heterogeneity and the absence of head-to-head comparisons preclude formal ranking. Gastrointestinal adverse events were frequent with glucagon-like peptide-1 (GLP-1) receptor agonists. Pediatric trials and real-world evidence indicate partial weight regain after discontinuation, while prescribing remains limited and unequal. Conclusions: Pediatric obesity care should be phenotype-informed, multidisciplinary, and longitudinal. Pharmacotherapy can enhance BMI reduction but should complement rather than replace behavioral, psychological, and family care. Outcomes should extend beyond BMI to metabolic, psychological, functional, and long-term measures.

## 1. Introduction

Childhood obesity is a widespread global health issue [1]. When obesity is not secondary to endocrine, monogenic, or syndromic disorders, it can be considered ‘essential’ obesity: a chronic, relapsing, and multifactorial disease arising from interactions among biological susceptibility, eating behavior, environmental exposures, and socioeconomic conditions. In children and adolescents, body mass index (BMI) must be interpreted by age and sex. For individuals aged 5–19 years, the World Health Organization (WHO) defines overweight as BMI-for-age greater than one standard deviation above the reference median and obesity as greater than two standard deviations; comparisons across studies must nevertheless account for differences between WHO and International Obesity Task Force classification systems [1,2].

The global burden has increased sharply. In 2022, more than 390 million people aged 5–19 years were living with overweight, including approximately 160 million with obesity, and the prevalence of overweight in this age group had risen from about 8% in 1990 to 20% in 2022 [1,3]. In the sixth round of the WHO European Childhood Obesity Surveillance Initiative, 25% of children aged 7–9 years were living with overweight, including 11% with obesity [4]. Italian surveillance similarly indicates that approximately three in ten children aged 8–9 years have excess weight, with higher prevalence in southern regions [5]. Socioeconomic inequalities are marked: analysis of 53,275 Italian children showed less favorable dietary and activity patterns and higher obesity prevalence among children from families with lower parental education [6].

Pediatric obesity is associated with hypertension, insulin resistance, type 2 diabetes, dyslipidemia, metabolic dysfunction-associated steatotic liver disease, obstructive sleep apnea, and other early or adult complications. It also frequently persists into adulthood and predicts later cardiometabolic morbidity and premature cardiovascular mortality [7,8,9,10]. Its burden is also psychological and social: weight stigma, bullying, body dissatisfaction, anxiety or depressive symptoms, impaired quality of life, and disordered eating may occur, although these outcomes are heterogeneous and should not be assumed in every child [11,12,13]. Neurodevelopmental comorbidity is relevant in selected patients. Attention-deficit/hyperactivity disorder (ADHD) is associated with a modest increase in the risk of pediatric overweight and obesity; reward-related behavior may overlap between the two conditions, whereas cognitive-control difficulties appear more specific to ADHD [14,15,16].

Given its complex pathogenesis and clinical expression, treatment cannot be reduced to dietary prescription. Contemporary care combines nutrition, physical activity, behavioral strategies, psychological support, and family involvement, while accounting for treatment accessibility and social context [10,17]. Recently, the therapeutic landscape has also expanded: while intensive nutritional, behavioral, and family-based interventions remain foundational, newer drugs such as liraglutide, semaglutide, and other anti-obesity medications can produce rapid and clinically meaningful BMI reductions in selected young people. Their long-term safety, durability after discontinuation, accessibility, and integration with psychological and family care remain incompletely defined [18,19,20]. The aim of the present review is to discuss recent evidence on the integration of psychological, family-based, and pharmacological approaches to pediatric obesity, with a focus on treatment engagement, eating psychopathology, and the more limited evidence on the relationship between obesity, ADHD, and stimulant-associated weight trajectories. These domains were selected because each can influence whether a child engages in treatment, how the child responds, and whether the response is maintained: family resources and socioeconomic barriers affect access and attendance; psychopathology, disordered eating, and neurodevelopmental disorders may modulate engagement and response; and pharmacotherapy raises new questions about durability after discontinuation.

## 2. Materials and Methods

This narrative review was supported by a structured literature search designed to identify studies directly relevant to the multidisciplinary management of pediatric obesity, with particular attention to psychological and family-related factors, eating psychopathology, neurodevelopmental comorbidities, treatment engagement, and pharmacological approaches. Searches were conducted between April and August 2026 through the University of Turin library discovery platform, a single search interface that covers PubMed/MEDLINE, Scopus, and Web of Science; the three databases were therefore not searched separately. The searches were supplemented by backward and forward citation tracking of key publications. The search primarily covered studies published from 2010 to August 2026, with particular emphasis on recent evidence from 2015–2026, while retaining earlier landmark studies when necessary to establish relevant epidemiological, clinical, neurobehavioral, or therapeutic findings.

Search terms were used in different combinations and included “pediatric obesity”, “childhood obesity”, “adolescent obesity”, “overweight”, “multidisciplinary treatment”, “lifestyle intervention”, “behavioral intervention”, “family-based treatment”, “psychological intervention”, “cognitive behavioral therapy”, “motivational interviewing”, “emotional regulation”, “treatment adherence”, “attrition”, “socioeconomic factors”, “telehealth”, “depression”, “anxiety”, “weight stigma”, “body dissatisfaction”, “eating disorders”, “binge eating”, “loss-of-control eating”, “ADHD”, “attention-deficit/hyperactivity disorder”, “executive function”, “impulsivity”, “inhibitory control”, “delay discounting”, “reward processing”, “neuroimaging”, “stimulant treatment”, “methylphenidate”, “lisdexamfetamine”, “anti-obesity medication”, “GLP-1 receptor agonist”, “liraglutide”, “semaglutide”, “orlistat”, and “phentermine/topiramate”. The main searches combined “obesity” with “childhood”, “treatment”, “pharmacotherapy”, and “incidence”, and were then refined by combining “pediatric obesity” or “childhood obesity” with the other terms listed above. Additional targeted searches were conducted when specific clinical, neurobehavioral, treatment-related, or pharmacological questions emerged during manuscript development.

Priority was given to peer-reviewed studies directly examining pediatric obesity and its treatment, including randomized controlled trials, prospective and retrospective cohort studies, large observational and real-world studies, and clinically relevant cross-sectional investigations. Systematic reviews, meta-analyses, and international clinical guidelines were used to characterize the broader evidence base, identify relevant primary studies, and provide context for areas supported by a large or heterogeneous literature. Official reports from recognized public-health institutions, including the World Health Organization and the Italian National Institute of Health, were also considered for epidemiological and surveillance data, and current U.S. prescribing information was consulted during manuscript revision for the indications, contraindications, and safety warnings of anti-obesity medications.

Studies involving children and adolescents younger than 18 years were prioritized. Studies including both pediatric and adult participants were retained when pediatric findings were reported separately or when they provided information directly relevant to the aims of the review. Adult-only studies were generally excluded from the main evidence synthesis; however, selected adult studies were retained solely as contextual evidence when controlled pediatric data were unavailable, including studies on binge-eating disorder treated with lisdexamfetamine, post-treatment weight trajectories after semaglutide withdrawal, and family involvement in the treatment of anorexia nervosa. Findings derived from adult populations were interpreted as indirect or hypothesis-generating evidence and were not considered evidence of efficacy or safety in children and adolescents. The limited pediatric observational evidence on stimulant treatment and loss-of-control eating was considered separately and was not treated as evidence supporting routine clinical use.

Studies focusing primarily on monogenic or syndromic obesity were not included unless they provided information directly applicable to the broader clinical questions addressed in the review. Duplicate publications, conference abstracts, non-peer-reviewed sources (other than the institutional and regulatory documents described above), and studies without a direct connection to pediatric obesity, its psychological or neurodevelopmental correlates, treatment engagement, or therapeutic management were excluded. Narrative reviews were used mainly to identify relevant concepts and primary studies rather than as the principal source for quantitative estimates whenever original evidence was available. Both authors assessed the relevance and eligibility of the retrieved publications. Disagreements were resolved through discussion and consensus.

## 3. Results

A total of 57 sources were included in the narrative synthesis: 12 randomized or controlled clinical trials, 15 observational studies, 14 systematic reviews or meta-analyses, 8 narrative reviews or editorials, 1 clinical practice guideline, 3 public-health reports, and 4 drug prescribing information documents. The findings are presented below by thematic area.

### 3.1. Multicomponent Lifestyle and Behavioral Interventions

Intensive health behavior and lifestyle treatment combines nutritional counseling, physical activity, reduced sedentary behavior, and behavioral techniques, usually with family participation. The 2023 American Academy of Pediatrics (AAP) guideline recommends family-based, multicomponent treatment and identifies 26 or more face-to-face contact hours over a 3- to 12-month period as the most effective intensity [10,21]. In the technical evidence review supporting the guideline, Skinner and coworkers included 215 intervention studies; among the 126 randomized trials of lifestyle or dietary interventions, psychosocial outcomes were reported in only about 20% and mental-health outcomes in 5%, confirming that the evidence supporting clinical guidelines remains predominantly weight-centered [21].

In preschool-aged children, more intensive multidisciplinary interventions appear to improve anthropometric outcomes [22]. Effective programs commonly use self-monitoring, goal setting, problem solving, environmental restructuring, modeling, and relapse-prevention strategies. A systematic review found that nine obesity-management interventions used a mean of 7.5 behavior-change techniques, but specific psychological components, such as emotion-regulation training and motivational interviewing, were rarely included. Moreover, intervention effectiveness was not explained simply by the total number of behavior-change techniques used, suggesting that their selection, implementation, and adaptation to the child and family may be more important than their quantity alone [23].

### 3.2. Psychological and Family-Based Interventions

Evidence from a large randomized clinical trial supports family-based behavioral treatment. In 452 children aged 6–12 years, a primary-care intervention targeting eating, physical activity, and parenting practices produced a greater reduction in percentage above median BMI than usual care at 24 months (between-group difference −6.21 percentage points, 95% confidence interval [CI] −10.14 to −2.29); benefits also extended to participating parents and to untreated siblings with overweight or obesity [24]. In the smaller two-arm ENTREN/ENTREN-F trial (ENTREN-F, *n* = 30; ENTREN, n = 40), both intervention protocols included nutritional counseling, support for physical activity, emotional-regulation training, and work on social relationships, using cognitive-behavioral and motivational techniques. Adding parallel sessions for parents was associated with better adherence, greater reductions in BMI z-score and child anxiety, and greater family adaptability, although the sample of 70 children limits definitive conclusions [25]. The main family-based and psychological intervention studies discussed in this review are summarized in Table 1.

The pragmatic TEAM UP trial extended these findings to 730 children and adolescents across 41 primary-care practices. At 12 months, enhanced standard care plus family-based behavioral treatment reduced percent median BMI more than enhanced standard care alone (between-group difference −3.8 units, 95% CI −6.20 to −1.34; *p* = 0.002). The study included substantial socioeconomic diversity, with 47% of participants insured by Medicaid and 22% reporting household food insecurity, supporting the feasibility of family-based treatment in routine primary care [26].

### 3.3. Treatment Engagement and Feasibility

Participation in pediatric obesity treatment can be limited by family members’ time constraints (parents’ work and childcare, and children’s school commitments), transportation, financial costs, and the complexity of treatment information. A systematic review of 26 studies on the recruitment of children into nutrition, physical activity, and obesity intervention trials identified time constraints, the complexity of trial information, and transportation or lack of childcare as the main barriers, whereas perceived benefits, compensation or incentives, physician recommendation, and support were the most frequent facilitators [27]. In the subsequent three-arm randomized ENTREN-F trial, mean attendance was 72.4% in ENTREN-F and 68.2% in ENTREN, versus 54.9% in the brief control condition (overall *p* = 0.001); the control group attended significantly less than both ENTREN (*p* = 0.017) and ENTREN-F (*p* = 0.001), while the two multidisciplinary programs did not differ significantly [28]. Lower attendance was also associated with lower maternal education, higher maternal depressive symptoms, more maternal critical comments toward the child, and greater child weight severity; logistical barriers accounted for 47.9% of reported attrition reasons [28].

All these barriers intersect with socioeconomic inequalities in obesity risk [6]. Since logistical difficulties were the most frequently reported barriers, remote or hybrid care may reduce treatment burden, although evidence remains preliminary and engagement may still be limited by less readily recognized psychological factors. In an Italian telehealth program, 117 of 156 eligible patients (75.0%) participated during the COVID-19 lockdown; 75 participants (64.1%) returned for post-lockdown assessment, compared with only 7 of 39 nonparticipants (17.9%). This marked differential follow-up and the very small comparison group limit conclusions to feasibility rather than comparative efficacy [29].

### 3.4. Psychopathology and Disordered Eating

Psychiatric comorbidity and eating disorders, particularly binge-eating disorder (BED), are clinically relevant in pediatric obesity but have heterogeneous presentations and frequently co-occur. In a sample of 170 Spanish children aged 8–12 years with overweight or obesity, recruited through primary-care centers and schools, all participants underwent a standardized diagnostic interview and questionnaires assessing anxiety, depression, and disordered eating. Overall, 57.1% of participants met criteria for at least one psychiatric disorder, and 33.5% reported loss-of-control eating [30]. The authors cautioned that these estimates may be inflated, because participation was voluntary and families were aware of the diagnostic interview, and that they cannot be generalized to all children with overweight or obesity. Conversely, children without a categorical diagnosis may still present clinically relevant subthreshold symptoms. Symptoms such as emotional eating, eating in the absence of hunger, loss-of-control eating, binge eating, restrictive dieting, body dissatisfaction, and experiences of weight-related stigma often occur without meeting criteria for a specific Diagnostic and Statistical Manual of Mental Disorders (DSM) diagnosis and still require targeted assessment and tailored treatment [12].

Prospective evidence suggests possible bidirectional relationships between obesity and eating-disorder symptoms, whereas findings for depression vary by age and sex and evidence for anxiety remains insufficient [13]. In prospective studies, psychological outcomes are assessed with structured interviews or validated measures, but instruments and thresholds vary, and subthreshold symptoms may still interfere with eating behavior and obesity treatment. The absence of a categorical diagnosis should therefore not be equated with absence of clinically relevant symptoms [13]. Concern that obesity treatment might exacerbate eating psychopathology arises primarily from observational associations between self-reported dieting outside professionally supervised treatment and subsequent disordered-eating behaviors, rather than from evidence that professionally supervised dietary treatment causes eating disorders [31]. Jebeile et al. synthesized 36 pediatric obesity-treatment studies involving 2589 participants. All interventions combined a dietary component with behavior-modification strategies; some also addressed eating-disorder-related content, eating behaviors, or body image, and a psychologist, counselor, or therapist was involved in 17 studies. At post-treatment, binge eating (standardized mean difference [SMD] −0.588, *p* < 0.001), bulimic symptoms (SMD −0.326, *p* < 0.001), emotional eating (SMD −0.149, *p* = 0.008), and drive for thinness (SMD −0.167, *p* = 0.005) decreased; overall eating-disorder risk was also reduced at follow-up (SMD −0.313, *p* = 0.012). Moderator analyses found no significant effect of psychologist involvement on change in eating-disorder risk, so the observed improvement cannot be attributed specifically to psychotherapy. Overall, structured and professionally supervised obesity treatment with a dietary component did not, on average, worsen eating psychopathology and improved several related symptoms. Continued monitoring remains appropriate because eating-disorder prevalence was reported in only five studies and participants who withdrew from treatment were poorly represented [31].

**Table 1:** Main family-based and psychological intervention studies discussed in this review.

Study	Design; Setting	Participants	Intervention	Comparator; Duration	Main Outcomes	Main Limitations
Epstein et al., 2023 [24]	RCT; 4 U.S. pediatric primary-care settings	452 children aged 6–12 y with overweight or obesity, their parents, and 106 siblings	Family-based behavioral treatment targeting eating, physical activity, and parenting, delivered by a trained coach; target of 26 sessions over 24 months, individualized to family progress	Usual care; 24 months	Child percentage above median BMI −6.21 (95% CI −10.14 to −2.29) vs. usual care; improvements also in parents and untreated siblings	U.S. setting only; weight outcomes primary; psychological outcomes not reported as primary endpoints
Staiano et al., 2026 (TEAM UP) [26]	Pragmatic RCT; 41 primary-care practices in 4 U.S. states	730 children aged 6–15 y; 46.6% insured by Medicaid, 22.1% with household food insecurity	Enhanced standard care plus family-based behavioral treatment: 26–33 sessions over 12 months (diet, physical activity, parenting, social support) by dietitians, social workers, or community health workers, in person or via telehealth	Enhanced standard care by primary-care practitioners; 12 months	Percent median BMI −3.8 units (95% CI −6.20 to −1.34; *p* = 0.002) vs. enhanced standard care	Mean attendance 16.7 sessions; lower engagement among Medicaid-insured and food-insecure families; COVID-19 disruptions; 12-month follow-up
Sepúlveda et al., 2020 (ENTREN/ENTREN-F) [25]	Two-arm trial; primary care, Madrid, Spain	70 children aged 8–12 y with overweight or obesity (ENTREN-F, n = 30; ENTREN, n = 40)	ENTREN: 12 two-hour sessions over 6 months (nutrition, physical activity, emotional regulation, cognitive-behavioral and motivational techniques); ENTREN-F adds six parallel parent sessions on family environment and communication	ENTREN without family component; 6 months + 6-month follow-up	ENTREN-F: higher adherence, greater BMI z-score reduction, lower child anxiety, and higher family adaptability than ENTREN	Small sample; one recruitment wave not randomized
Rojo et al., 2022 (ENTREN-F RCT) [28]	Three-arm RCT; primary care, Madrid, Spain	232 families of children aged 8–12 y with overweight or obesity assessed at baseline	ENTREN-F and ENTREN programs (as above)	Brief standard behavioral intervention (4 sessions); 6 months	Attendance 72.4% (ENTREN-F), 68.2% (ENTREN), 54.9% (control); attrition 27.4%, 38.5%, 66.7%; logistical barriers were the most frequent reason for attrition (47.9%)	Short-term adherence outcomes only; high attrition in the control arm
Pecoraro et al., 2021 [29]	Retrospective observational; outpatient family-based obesity program, southern Italy	156 children and adolescents with obesity (mean age ≈ 11 y)	Telehealth during COVID-19 lockdown: video calls with each member of the multidisciplinary team, home exercise, and age-adapted tutorials in a closed social group	Non-participants (n = 39); lockdown period	117/156 (75.0%) participated; post-lockdown follow-up 64.1% vs. 17.9%; BMI z-score did not increase in either group; fat-free mass increased among participants	Retrospective design; very small comparison group at follow-up (n = 7); differential follow-up
Jebeile et al., 2019 [31]	Systematic review and meta-analysis	36 studies; 2589 participants aged 7.8–16.9 y	Obesity treatment with a dietary component combined with behavioral strategies; psychologist, counselor, or therapist involved in 17 studies	Pre–post comparisons; follow-up 6 months to 6 years	Post-treatment reductions in binge eating (SMD −0.59), bulimic symptoms (−0.33), emotional eating (−0.15), and drive for thinness (−0.17); eating-disorder risk reduced at follow-up (−0.31)	Eating-disorder prevalence reported in only five studies; participants who withdrew poorly represented

Abbreviations: BMI, body mass index; CI, confidence interval; RCT, randomized controlled trial; SMD, standardized mean difference.

### 3.5. ADHD and Related Neurobehavioral Considerations

Among neurodevelopmental conditions, ADHD deserves specific attention. Two meta-analyses found a modest but consistent association between ADHD and pediatric overweight or obesity (pooled odds ratios 1.20 and 1.56), and the relationship should not be overstated [14,15]. Neurodevelopmental disorders may be concentrated among children seeking specialist obesity treatment: in two outpatient obesity clinics, 47 of 80 children aged 8–17 years had at least one neurodevelopmental disorder, 17 of whom were diagnosed only after study screening; however, referral and selection effects prevent extrapolation to all children with obesity [32]. ADHD may also influence treatment response: among 456 youths treated in a pediatric weight-management clinic, BMI decreased more slowly in those with ADHD than in those without, whereas youths with ADHD receiving stimulant medication showed a faster BMI decrease than those not receiving it [33]. ADHD screening is therefore reasonable in children with obesity when attentional or organizational difficulties, school impairment, impulsivity, or repeated failure to follow structured recommendations persist despite adequate support; a positive screen must be followed by a full diagnostic assessment.

Regarding mechanisms, current evidence supports partly shared but distinguishable pathways in obesity and ADHD. Reward sensitivity and reward-related decision-making may overlap in ADHD and overweight/obesity, whereas impaired cognitive control appears more specifically associated with ADHD [16]. Dopaminergic and noradrenergic signaling provides a plausible neurobiological link, but current evidence does not support a single shared mechanism: in a structural neuroimaging study of 471 children aged 8–12 years, ADHD was associated mainly with reduced cortical surface area and overweight/obesity mainly with reduced cortical thickness in frontal regions, with only partial overlap [34]. Clinically, shorter goals, immediate non-food reinforcement, reminders, predictable routines, and parental scaffolding are plausible adaptations for executive-function difficulties, although they have not been specifically validated in randomized obesity trials involving children with ADHD.

Stimulant medications are indicated for ADHD when the diagnosis has been confirmed by a comprehensive assessment; they are not anti-obesity medications, and their effects on weight should be regarded as effects of ADHD treatment that require monitoring. Observational and meta-analytic data suggest small early reductions in weight or BMI during stimulant treatment, with a need for individualized growth and nutritional monitoring; longitudinal data also raise the possibility of later BMI rebound [33,35,36]. In a retrospective review of 330 children and adolescents with ADHD, BMI z-score decreased by 0.41 in those with severe obesity and by 0.44 in those with mild-to-moderate obesity during the two years after starting lisdexamfetamine, with larger reductions in younger children; the authors suggested that this effect deserves further study in children with obesity, although the absence of a control group precludes causal inference [37]. ADHD is also associated with eating disorders: in a meta-analysis, individuals with ADHD had a higher risk of any eating disorder (odds ratio [OR] 3.82, 95% CI 2.34–6.24) and of binge-eating disorder (OR 4.13, 95% CI 3.00–5.67), and the association remained significant in pediatric samples (OR 3.59, 95% CI 1.51–8.50) [38]. The possible effect of stimulants on disordered eating is clinically relevant but remains preliminary. In a retrospective chart review of 25 adolescents aged 12–19 years with BED treated with lisdexamfetamine, 15 reported some improvement in BED symptoms, but BMI percentile did not decrease significantly [39]. In a prospective uncontrolled study of 10 children aged 8–13 years with ADHD and loss-of-control eating who started methylphenidate or lisdexamfetamine, mean loss-of-control episodes decreased from 10.3 to 2.1 over three months, with improvement reported in nine participants [40]. Both studies are very small and uncontrolled, and their findings should be interpreted with caution. In adults with BED, evidence that cannot be directly extrapolated to children shows that lisdexamfetamine reduces binge-eating frequency and that continued treatment lowers the risk of relapse (3.7% vs. 32.1% with placebo), outcomes distinct from weight maintenance [41,42]. These signals justify controlled pediatric trials but not the systematic use of stimulants as weight-loss treatment. Such trials should separately assess effects on ADHD symptoms, loss-of-control eating, weight, growth, cardiovascular safety, and misuse risk.

### 3.6. Pharmacological Treatment

Pharmacotherapy for obesity is increasingly used as an adjunct to behavioral treatment, especially in adolescents with more severe obesity unresponsive to dietary and behavioral treatment. The main pediatric placebo-controlled trials are summarized in Table 2. Torbahn and coworkers summarized an updated Cochrane systematic review evaluating the benefits and harms of anti-obesity medications in children and adolescents. The systematic review included 35 randomized trials involving 4331 participants; 25 trials involving 3088 participants contributed to the BMI meta-analysis. Compared with the same behavioral intervention with or without placebo, pharmacotherapy produced an overall mean difference in BMI of −1.71 kg/m^2^ (95% CI −2.27 to −1.14). Drug-specific subgroup estimates were −5.88 kg/m^2^ for semaglutide, −4.57 kg/m^2^ for phentermine/topiramate, −1.70 kg/m^2^ for sibutramine, −1.58 kg/m^2^ for liraglutide, −1.27 kg/m^2^ for metformin, −1.00 kg/m^2^ for exenatide, and −0.79 kg/m^2^ for orlistat. However, these estimates were derived from separate and heterogeneous trials rather than head-to-head comparisons; semaglutide and phentermine/topiramate were each represented by only one trial. Therefore, the larger numerical reductions observed with these two agents should not be interpreted as a formal comparative ranking [43,44]. Treatment effects varied substantially across agents, and evidence regarding long-term safety, weight regain, use in younger children, and underrepresented populations remained limited. Older pharmacological approaches produced comparatively modest effects in individual trials. Six months of metformin reduced BMI by 1.09 kg/m^2^ compared with placebo in children with obesity and insulin resistance [45]. In a one-year randomized trial involving 539 adolescents, orlistat reduced BMI by 0.55 kg/m^2^, whereas BMI increased by 0.31 kg/m^2^ with placebo (*p* = 0.001). A BMI reduction of at least 5% was achieved by 26.5% of participants receiving orlistat versus 15.7% receiving placebo, while reductions of at least 10% occurred in 13.3% versus 4.5%, respectively. Gastrointestinal adverse events were generally mild to moderate; individual gastrointestinal events occurred in 9–50% of participants receiving orlistat versus 1–13% of those receiving placebo [46]. Among newer pharmacological options, placebo-controlled trials reported larger percentage reductions in BMI, although differences in study design preclude direct comparisons between agents. In adolescents, liraglutide produced a placebo-adjusted difference in the percentage change in BMI of −4.64 percentage points at 56 weeks, whereas once-weekly semaglutide produced a difference of −16.7 percentage points at 68 weeks [18,19]. Phentermine/topiramate produced placebo-adjusted differences of −8.11 percentage points with the mid dose (7.5/46 mg) and −10.44 percentage points with the top dose (15/92 mg) at 56 weeks [47]. Gastrointestinal adverse events were frequent with both glucagon-like peptide-1 (GLP-1) receptor agonists. With liraglutide, they occurred in 64.8% of participants versus 36.5% with placebo and mainly included nausea (42.4% vs. 14.3%), vomiting (34.4% vs. 4.0%), and diarrhea (22.4% vs. 14.3%). With semaglutide, gastrointestinal events occurred in 62% versus 42%, including nausea (42% vs. 18%), vomiting (36% vs. 10%), diarrhea (22% vs. 19%), and abdominal pain (15% vs. 6%). These events were generally mild or moderate and occurred mainly during or shortly after dose escalation. Cholelithiasis was reported in 4% of participants receiving semaglutide and in none receiving placebo [18,19]. Evidence in younger children is emerging: in a trial involving 82 children aged 6 to <12 years, liraglutide produced a 7.4-percentage-point greater reduction in BMI than placebo at 56 weeks [48].

Despite the promising evidence regarding newer anti-obesity medications, important uncertainties remain, particularly concerning the maintenance of weight reduction after treatment discontinuation. Direct pediatric evidence is available from randomized trials of liraglutide. In the trial involving 251 adolescents, liraglutide produced a placebo-adjusted reduction in BMI of 4.64 percentage points after 56 weeks of treatment. However, during the subsequent 26-week off-treatment period, BMI standard-deviation scores increased significantly more among participants previously receiving liraglutide than among those previously receiving placebo (estimated difference 0.15; 95% CI 0.07 to 0.23), indicating partial loss of the treatment effect after discontinuation [18]. A similar pattern was observed in the trial involving 82 children aged 6 to <12 years. At week 56, the estimated treatment difference in BMI change was −7.4 percentage points (−5.8% with liraglutide vs. +1.6% with placebo). During the following 26 weeks without treatment, BMI and body weight increased in both groups: between weeks 56 and 82, the observed mean change in BMI from baseline went from −6.7% to −0.8% in the liraglutide group and from +2.1% to +6.7% in the placebo group, suggesting that weight regain occurred but that part of the treatment benefit persisted [48].

These controlled findings are supported by real-world pediatric data. A large retrospective study conducted in Israel examined 307,208 children and adolescents aged 10–18 years with overweight or obesity, of whom only 2236 (0.7%) were prescribed metformin, liraglutide, or orlistat [49]. Among the 1717 participants with at least two medication dispensations, metformin—used off-label for obesity—was the most frequently prescribed medication (73.8%), followed by liraglutide (24.5%) and orlistat (1.7%). Median treatment duration was approximately 1.5–1.7 years for metformin and approximately 0.6 years for liraglutide and orlistat. Semaglutide was not among the medications analyzed. Overall, 55.1% of treated participants achieved a maximal reduction in BMI z-score greater than 0.2 standard deviations, with no significant differences among metformin, liraglutide, and orlistat (53.9%, 57.3%, and 58.3%, respectively; *p* = 0.588). Treatment was also associated with reductions in blood glucose, glycated hemoglobin (HbA1c), triglycerides, and total cholesterol and with an increase in high-density lipoprotein (HDL) cholesterol. After treatment discontinuation, BMI z-scores and cardiometabolic parameters partially regressed toward pretreatment values, although they remained significantly improved compared with baseline. These observational findings confirm that post-treatment weight regain can occur in routine pediatric practice while also suggesting that some anthropometric and metabolic benefits may persist. However, they do not establish long-term durability and cannot be directly extrapolated to newer agents, particularly semaglutide [49].

The proportion of adolescents with obesity prescribed an obesity medication approved by the U.S. Food and Drug Administration (FDA) increased from 0.1% in 2020 to 0.5% in 2023, corresponding to an adjusted increase of approximately 302% [50]. The medications considered were orlistat, phentermine, phentermine/topiramate, setmelanotide, high-dose liraglutide, and high-dose semaglutide, rather than GLP-1 receptor agonists alone. In 2023, semaglutide was the most frequently prescribed medication, accounting for 57.1% of treated adolescents, followed by phentermine or phentermine/topiramate (37.7%) and liraglutide (11.9%). Despite the relative increase, treatment reached only 0.5% of adolescents with obesity. Moreover, Black adolescents were 39% less likely than White adolescents to receive a prescription, even though severe obesity was more prevalent among them. High out-of-pocket costs, insurance restrictions, medication shortages, and differences in prescribing practices may contribute to this limited and unequal access [50]. Overall, available trials demonstrate increasingly large short-term reductions in BMI, but evidence remains limited regarding weight maintenance after treatment withdrawal, long-term safety across growth and puberty, equitable access, and the integration of pharmacotherapy with sustained psychological and family-based care [20,43].

## 4. Discussion

The treatment of pediatric obesity remains a challenge for public health and social welfare. New therapeutic tools are now available, with moderate-to-good evidence of efficacy [10,18,19,20,21,24,25,26,43,44,45,46,47,48,49,50]. Nevertheless, concerns persist regarding the short- and long-term effectiveness of treatment protocols, particularly pharmacological treatments, as well as logistical, economic, and psychological barriers [20,25,27,28,29,43,49,50]. Intensive lifestyle and behavioral interventions remain foundational, but their effectiveness is shaped by psychological functioning, eating behavior, family organization, socioeconomic resources, neurodevelopmental characteristics, and treatment accessibility [21,22,23,24,25,26,27,28,29,30]. Pharmacotherapy has produced substantially larger average BMI reductions, yet it does not replace the need for long-term, multidisciplinary care [10,20,43,44].

Beyond the persisting need to improve treatment effectiveness, availability, and adherence, and to integrate the available treatments across disciplines, some general issues emerge from the current review.

### 4.1. Beyond a Predominantly Weight-Centered Model

The first is a methodological issue: the available literature remains disproportionately focused on anthropometric outcomes. In the evidence review supporting the AAP guideline, psychosocial and mental-health outcomes were reported far less often than weight-related outcomes, despite the behavioral framing of most interventions [21]. BMI change is clinically important, but it does not capture body image, emotional regulation, eating psychopathology, family functioning, social participation, or quality of life. These domains are critical during development and may influence the child’s development independently of the physical consequences of excess weight. Conversely, improvement in these domains may be meaningful even when short-term BMI change is modest. A child whose BMI decreases while loss-of-control eating, depression, or major family and treatment burdens remain unchanged may have achieved an anthropometric response without a complete or durable therapeutic response.

Emotional symptoms may not be spontaneously recognized or reported by children and can be expressed through developmental changes in irritability, withdrawal, sleep, interests, school functioning, or eating behavior. Assessment should therefore integrate the child’s account, caregiver observations, and clinical evaluation, with specialist assessment when clinically indicated [10,17]. This broader framework should not be interpreted as an indication for psychotherapy in every child with obesity. Anxiety, depressive symptoms, body dissatisfaction, and disordered eating are more frequent in some clinical subgroups but are neither universal nor necessarily caused directly by adiposity [13,17]. The available meta-analytic evidence suggests that loss-of-control eating is particularly relevant because it describes the child’s relationship with food more directly than BMI and may indicate a need to address emotional regulation or eating psychopathology. The findings of Jebeile and coworkers challenge the assumption that professionally supervised dietary treatment necessarily worsens eating pathology: on average, several disordered-eating symptoms improved during and after treatment [31]. This suggests that a structured nutritional approach may also help to address impulsive eating and to support emotion management that does not rely on food intake. Psychopathological screening and longitudinal psychological monitoring should therefore accompany treatment, without allowing a generalized fear of triggering an eating disorder to prevent access to appropriate obesity care.

### 4.2. Family Context and Treatment Feasibility

Family-based trials show that caregivers can support changes in meal routines, food availability, reinforcement, physical activity, and environmental cues, with benefits demonstrated in both specialist and primary-care settings [24,25,26]. Although evidence from anorexia nervosa is not direct evidence for pediatric obesity, it reinforces the broader principle that family involvement should be framed as a therapeutic resource rather than as attribution of causal responsibility for the child’s weight [51].

Engagement depends partly on whether families can attend the scheduled contacts and translate recommendations into everyday routines. Time, transportation, work schedules, childcare, cost, parental distress, and socioeconomic disadvantage can reduce attendance and the treatment dose received [27,28]. Repeated non-attendance should therefore be treated as clinical information about a possible mismatch between treatment demands and family resources, rather than as automatic evidence of poor motivation. Flexible scheduling, simplified plans, delivery through primary care, and remote or hybrid follow-up may improve access. Telehealth data support feasibility, but current evidence is insufficient to regard remote treatment as universally equivalent to face-to-face multidisciplinary care [29].

### 4.3. Pharmacotherapy Within Multidisciplinary Care

Across separate placebo-controlled trials, semaglutide, liraglutide, and phentermine/topiramate produced larger average BMI reductions than older agents, although the absence of head-to-head comparisons precludes a formal ranking [18,19,43,44,47]. Nevertheless, greater efficacy should not create a new therapeutic reductionism. Medication can alter appetite, satiation, and energy intake but does not directly resolve weight stigma, family conflict, depression, treatment barriers, or disordered eating. Response to anti-obesity medication is highly heterogeneous: in the pivotal adolescent trials, a BMI reduction of at least 5% was achieved by 43% of participants receiving liraglutide and 76% of those receiving semaglutide [18,19]. Post hoc analyses suggest better responses to semaglutide in girls, younger adolescents, and those with lower baseline BMI, but reliable predictors of response are lacking [43]. The typical trajectory consists of a rapid BMI reduction followed by a plateau after about 6–12 months [43]. These data support offering medication to adolescents with obesity as an adjunct to intensive behavioral treatment, as recommended by current guidelines [10], with periodic reassessment of the individual response.

Age, eligibility, and safety should guide each prescription. In the United States, orlistat, liraglutide, semaglutide, and phentermine/topiramate are approved for adolescents aged 12 years or older (approval status in the European Union is summarized in Table 2), whereas phentermine alone is approved only from 16 years and for short-term use [43,52]. The AAP recommendation applies to adolescents with a BMI at or above the 95th percentile [10], and medication is often considered when lifestyle treatment alone has not been sufficient [52]; for liraglutide, the adolescent indication also requires a body weight above 60 kg [53]. Before prescribing, clinicians should review comorbidities, current medications that may promote weight gain, and contraindications [52]. Liraglutide and semaglutide are contraindicated in patients with a personal or family history of medullary thyroid carcinoma or multiple endocrine neoplasia type 2, and their labeling warns of pancreatitis, gallbladder disease, and suicidal behavior and ideation; liraglutide is contraindicated in pregnancy, and semaglutide should be discontinued at least 2 months before a planned pregnancy [53,54]. Phentermine/topiramate is contraindicated in pregnancy, glaucoma, hyperthyroidism, and during or within 14 days of monoamine oxidase inhibitor use; a negative pregnancy test is recommended before and monthly during treatment, and height velocity should be monitored because slower linear growth was observed in adolescents [55]. Orlistat is contraindicated in pregnancy, chronic malabsorption, and cholestasis, and a multivitamin containing fat-soluble vitamins is recommended [56]. Response should be reassessed after about 12 weeks at the maintenance dose, with discontinuation if the BMI reduction does not reach the threshold specified for each drug, and decisions to start, continue, or stop medication should be shared with the adolescent and the family [52,53,55].

The chronic nature of obesity is particularly evident after treatment cessation. In both pediatric liraglutide trials, part of the treatment effect was lost within 26 weeks of discontinuation, although mean BMI remained below baseline in children aged 6 to <12 years [18,48]; real-world data showed a similar partial regression of anthropometric and cardiometabolic improvements after medication cessation [49]. Contextual adult evidence from the STEP 1 extension strengthens this concern but cannot be directly extrapolated to pediatric patients: one year after semaglutide 2.4 mg and lifestyle intervention were withdrawn, participants had regained 11.6 percentage points, approximately two-thirds of the 17.3% mean weight loss achieved during treatment, and most cardiometabolic improvements had moved toward baseline [57]. Collectively, these findings are consistent with obesity as a chronic, relapsing disease and indicate that durability after withdrawal should be considered part of efficacy rather than a secondary concern [10,20,57]. A short medication course should therefore not be presented as a permanent solution. Moreover, medication may suppress appetite without modifying emotional distress, loss-of-control eating, family difficulties, or neurodevelopmental barriers that contribute to obesity maintenance; these unaddressed factors could limit treatment durability. Longer follow-up is needed to define safety across growth and puberty, treatment duration, discontinuation strategies, nutritional adequacy, body composition, and psychological effects.

Access is a further limitation, as prescribing remains uncommon and socially patterned (Section 3.6) [50].

### 4.4. Clinical Implications, Limitations, and Future Directions

The assessment of children with obesity should extend beyond BMI and metabolic complications to include eating patterns, loss-of-control eating, emotional symptoms, body dissatisfaction, weight-related victimization, family functioning, and practical barriers to treatment [10,12,13,17,21,27,30,31]. Because children may not spontaneously identify or report internalizing symptoms, assessment should be multi-informant and followed by specialist evaluation when indicated [10,13,17]. Neurodevelopmental evaluation should be targeted rather than universal and should never infer ADHD from obesity, overeating, or poor adherence alone [14,15,16,32,34]. When ADHD is confirmed and stimulant treatment is clinically indicated, appetite, nutritional intake, height, weight, BMI trajectory, and blood pressure should be monitored longitudinally [33,35,36]. By bringing together neuropsychiatric, family, and pharmacological perspectives that are often considered separately, this review highlights two points: psychopathology, disordered eating, and neurodevelopmental disorders may influence both engagement in treatment and response to it, and the durability of treatment effects after discontinuation should be regarded as part of efficacy.

The present review has limitations. Its narrative design does not provide the reproducibility or pooled estimates of a systematic review, and the included literature is heterogeneous in age, obesity definition, setting, intervention intensity, follow-up, and outcome selection. Evidence on psychological and neurodevelopmental phenotypes is often cross-sectional or observational, whereas pharmacological trials are generally shorter than the expected duration of obesity treatment. Pediatric stimulant studies involving loss-of-control eating are uncontrolled and extremely small, while adult BED findings remain translational; neither can establish pediatric treatment efficacy.

Future trials should determine which combinations of behavioral, family, psychological, and pharmacological components work for specific clinical phenotypes. Studies involving obesity and ADHD should prospectively evaluate treatment engagement, executive functioning, eating behavior, growth, body composition, and cardiometabolic outcomes rather than relying on post hoc BMI analyses. Pharmacotherapy studies require longer follow-up through puberty and after discontinuation and should include patients with psychiatric and neurodevelopmental comorbidity who more closely resemble routine clinical populations. The central research question is no longer simply which intervention reduces BMI, but which combination improves metabolic health, psychological well-being, functioning, and long-term participation for a particular child and family.

## 5. Conclusions

Intensive family-based, multicomponent behavioral treatment remains the best-supported foundation of pediatric obesity care, particularly when it is sufficiently intensive and feasible for the family. Pharmacotherapy can substantially increase and accelerate average BMI reduction in selected adolescents, but it should be integrated with rather than substituted for behavioral, psychological, and family care. Benefits may diminish after medication withdrawal, underscoring the chronic nature of obesity and the need for an explicit long-term treatment plan. Mental-health and eating-behavior assessment should be systematic without assuming that every child with obesity has a psychiatric disorder. In selected patients, unrecognized emotional distress, loss-of-control eating, ADHD, or executive-function difficulties may impair engagement or limit the durability of a weight-directed intervention. Recurrence is therefore more plausibly understood as the result of interacting biological, behavioral, environmental, and sometimes psychological mechanisms than as simple treatment failure or lack of motivation. Treatment success should extend beyond BMI to metabolic health, eating regulation, psychological well-being, family functioning, participation, and sustained outcomes. The most defensible model is phenotype-informed, multidisciplinary, and longitudinal.

## Figures and Tables

**Table 2:** Main pharmacological agents evaluated in placebo-controlled trials for the treatment of obesity in children and adolescents.

Drug (Mechanism)	Pivotal Pediatric RCT	Duration; Comparator	Main Weight Outcome vs. Comparator	Main Adverse Effects	Regulatory Status for Pediatric Obesity	Main Limitations
Metformin (biguanide; insulin sensitizer)	Yanovski et al., 2011 [45]; n = 100; 6–12 y; obesity with insulin resistance; single center	6 months (+6-month open-label phase); placebo + dietitian-led weight-reduction program	BMI −1.09 kg/m^2^; BMI z −0.07. Pooled estimate: −1.27 kg/m^2^ [44]	Gastrointestinal symptoms more frequent; limited the maximal tolerated dose in 17%	Not approved for obesity (off-label use); approved for type 2 diabetes from 10 y	Small, short, single-center trial; modest effect; selected insulin-resistant population
Orlistat (gastrointestinal lipase inhibitor)	Chanoine et al., 2005 [46]; n = 539; 12–16 y; multicenter	52 weeks; placebo + diet, exercise, and behavioral therapy	BMI −0.55 vs. +0.31 kg/m^2^; ≥5% BMI reduction 26.5% vs. 15.7%. Pooled estimate: −0.79 kg/m^2^ [44]	Gastrointestinal events (e.g., fatty/oily stool) in 9–50% vs. 1–13%; mostly mild to moderate	FDA: ≥12 y. EMA: adults only	About one-third did not complete the study; modest effect; poor gastrointestinal tolerability; no off-treatment follow-up
Liraglutide 3.0 mg daily (GLP-1 receptor agonist)	Kelly et al., 2020 [18]; n = 251; 12–<18 y	56 weeks + 26-week off-treatment follow-up; placebo + lifestyle therapy	BMI change −4.64 percentage points; ≥5% BMI reduction 43.3% vs. 18.7%. Pooled estimate: −1.58 kg/m^2^ [44]	Gastrointestinal events 64.8% vs. 36.5% (nausea 42.4%, vomiting 34.4%)	FDA and EMA: ≥12 y	Modest mean effect with heterogeneous response; greater BMI SDS increase than placebo after discontinuation (+0.15)
Liraglutide 3.0 mg daily (GLP-1 receptor agonist)	Fox et al., 2025 [48]; n = 82; 6–<12 y	56 weeks + 26-week off-treatment follow-up; placebo + lifestyle therapy	BMI change −7.4 percentage points	Gastrointestinal events 80% vs. 54%	EMA: 6–<12 y with body weight ≥45 kg (extension approved in 2025). FDA: ≥12 y only	Small sample; BMI increase after discontinuation; long-term effects on growth and puberty unknown
Semaglutide 2.4 mg weekly (GLP-1 receptor agonist)	Weghuber et al., 2022 [19]; n = 201; 12–<18 y	68 weeks + 7-week off-treatment follow-up; placebo + lifestyle therapy	BMI change −16.7 percentage points; ≥5% BMI reduction 76% vs. 23%. Pooled estimate: −5.88 kg/m^2^ [44]	Gastrointestinal events 62% vs. 42%; cholelithiasis 4% vs. 0%	FDA and EMA: ≥12 y	Single trial; 79% White participants; very short off-treatment follow-up; long-term safety through growth and puberty unknown
Phentermine/topiramate extended release (sympathomimetic + anticonvulsant)	Kelly et al., 2022 [47]; n = 227; 12–<17 y	56 weeks; placebo + lifestyle therapy	BMI change −8.11 (mid dose, 7.5/46 mg) and −10.44 (top dose, 15/92 mg) percentage points. Pooled estimate: −4.57 kg/m^2^ [44]	Overall adverse events similar to placebo; psychiatric events 7.4–8.8% vs. 1.8% (including depression)	FDA: ≥12 y. EMA: not authorized	Single industry-funded trial; no off-treatment follow-up; psychiatric monitoring and pregnancy prevention (topiramate) required

All trials added the drug to a behavioral or lifestyle intervention. Percentage-point differences refer to the placebo-adjusted percentage change in BMI from baseline. Pooled estimates are drug-specific mean differences in BMI (kg/m^2^) from the updated Cochrane review [44]; semaglutide and phentermine/topiramate were each represented by a single trial, and estimates from separate trials should not be interpreted as a head-to-head ranking. Abbreviations: BMI, body mass index; EMA, European Medicines Agency; FDA, U.S. Food and Drug Administration; GLP-1, glucagon-like peptide-1; RCT, randomized controlled trial; SDS, standard-deviation score.

## Data Availability

No new data were created or analyzed in this study. Data sharing is not applicable to this article.
